# Sarcomatoid Carcinoma of the Prostate: Ductal Adenocarcinoma and Stromal Sarcoma-Like Appearance: A Rare Association

**DOI:** 10.1155/2011/702494

**Published:** 2011-07-13

**Authors:** David Parada, Karla B. Peña, Francesc Riu

**Affiliations:** ^1^Department of Pathology, University Hospital Sant Joan, Reus, Tarragona 43201, Spain; ^2^Departamento de Anatomía Patológica, Hospital Universitario Sant Joan de Reus, Calle S/No. Reus, Tarragona, 43201, Spain

## Abstract

Sarcomatoid carcinoma (SC) of prostate gland is a rare biphasic tumour. In about half of cases, initial diagnosis is acinar adenocarcinoma, followed by nonsurgical therapy, with a subsequent diagnosis of SC. The survival rate is lower. We report a case of an 59-years-old man with unusual histopathologic finding of prostate sarcomatoid carcinoma, showing characteristics of ductal prostatic adenocarcinoma and prostatic stromal sarcoma-like appearance. Ductal adenocarcinoma was characterized by tall columnar cells with abundant amphophilic to eosinophil cytoplasm. Pleomorphic sarcoma was characterized to have overall glandular growth pattern, simulating a malignant phyllodes tumour. Estrogen and progesterone receptors showed nuclear immunostaining in mesenchymal multinucleated giant cells. In conclusion, SC of the prostate is an exceedingly rare tumour. Retrospective analyses render prostate SC as one of the most aggressive prostate malignancies. The prognosis is dismal regardless of other histologic or clinical findings.

## 1. Introduction

Sarcomatoid carcinoma (SC) of the prostate is a very rare neoplasia that display mixed epithelial and mesenchymal differentiation [1, 2]. There is considerable controversy in the literature regarding nomenclature and histogenesis of these tumours. Some authors use the term carcinosarcoma (CS) if there are heterologous components and sarcomatoid carcinoma if there are not [2, 3]. However, the most recent World Health Organization classification of urinary tract tumour does not distinguish between SC and CS and use the term “sarcomatoid carcinoma” to denote all of these lesions [1]. Clinically, most patients are 70 years old (range 50–89) and present with urinary tract obstruction and symptoms of frequency, urgency, and nocturia [4]. In about half of the cases, the initial diagnosis is acinar adenocarcinoma, followed by hormonal and/or radiation therapy, with a subsequent diagnosis of SC [5–9]. The mean time for the development of SC after acinar adenocarcinoma is 3 years [10, 11]. In published series, 5- and 7-year survivals were 41% and 14%, respectively [5, 6]. Another study showed that the actuarial risk of death at 1 year after diagnosis of sarcomatoid carcinoma was 20% [7].

We report the case of a 59-years-old man with unusual histopathologic findings of prostate sarcomatoid carcinoma, showing characteristics of ductal prostatic adenocarcinoma and prostatic stromal sarcoma-like appearance (homologous component). We also discuss the clinical, diagnostic, and therapeutic aspects of this uncommon tumour.

## 2. Case Report

A 59-year-old man presented to emergency with acute urinary retention and perineal pain in December 2006. He reported a previous history of hematuria and urinary retention and denied any constitutional symptoms. A history of frequent micturition, dysuria, poor urinary stream, and nocturia of approximately 12-month duration was also present. There was no family history of genitourinary cancer. No exposure to hazardous chemicals was confirmed.

**Figure 1:**
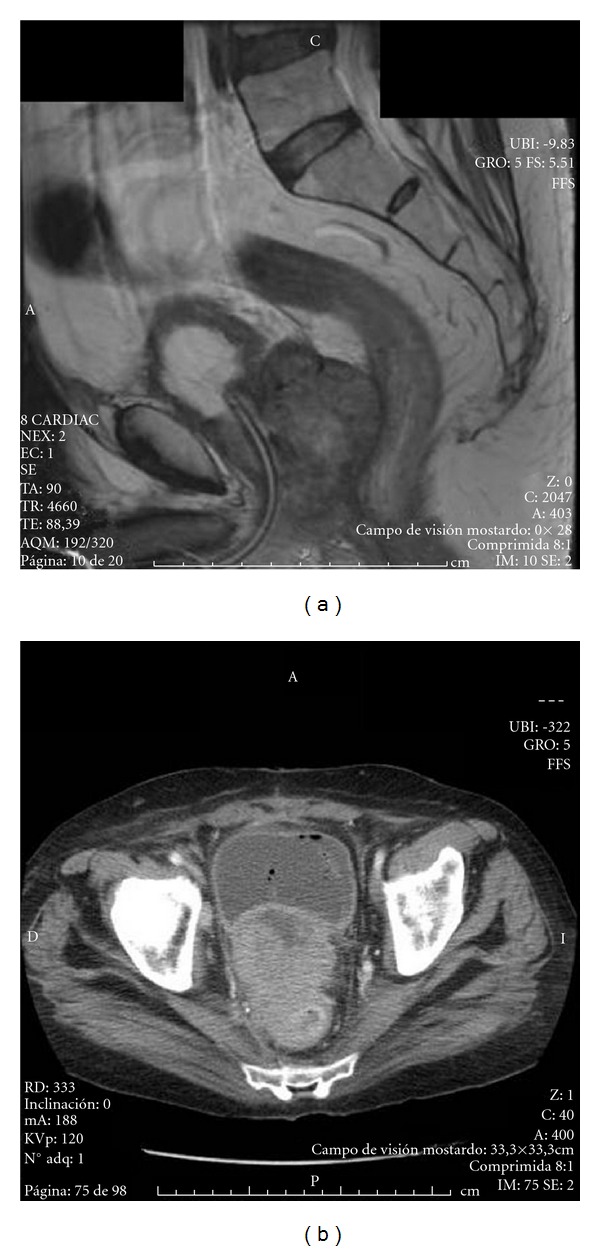
(a) Pelvic MRI considered suspicious for extracapsular extension of the tumour. (b) Pelvic TC scan showed tumour with extents hypodense zones, consistent with necrosis.

Rectal examination revealed a moderately enlarged normal prostate gland, consistent with prostatic adenoma grade 2/3. There was no palpable lymphadenopathy, and the rest of his physical examination was unremarkable. His last PSA, obtained one month earlier by his primary care physician as part of routine annual physical examination, was 1.49 ug/L. Since no pathology was found in both physical examination and laboratory tests, he underwent in January 2007 retropubic adenomectomy by Millin's technique without complications. Due to the unexpected findings of the pathology, the patient underwent further evaluation. Pelvic MRI considered suspicious for extracapsular extension of the tumour (Figure 1(a)) and lymph node affectation (extern iliac). Computed tomography of chest and brain as well as bone scan was negative for metastasis disease. The patient received adriamycin and ifosfamide and hormone therapy. Progression was confirmed in January 2008, and local radiotherapy was performed (39 Gy) with partial response (50%). In February 2009, hormone treatment was retired, and he received Taxotere without clinical response. In October 2010, a rectovesical fistula was confirmed by progression disease and uncontrolled lumbar pain (Figure 1(b)). A suprapubic and epidural catheters were collocated. The patient remained hospitalized to January 2011 with controlled pain and progression disease status.

## 3. Materials and Methods

The excised specimen was fixed in 10% buffered formalin and processed for routine histopathological study. Immunohistochemistry was performed on paraffin embedded tissue. The thin-sliced materials were immunostained by EnVision FLEX Kit and placed in a Dako Autostainer (EnVisionTM Systems, DAKO, Carpinteria, Calif, USA). For specific immunohistochemical details, see Table 1.

## 4. Results

### 4.1. Gross Pathology

Grossly, the biopsy consisted in varies irregulars fragments, weighed 40 gr. At cut sections there were elastic with grey-white areas. Haemorrhage and necrosis areas were observed.

### 4.2. Microscopic Findings

**Figure 2:**
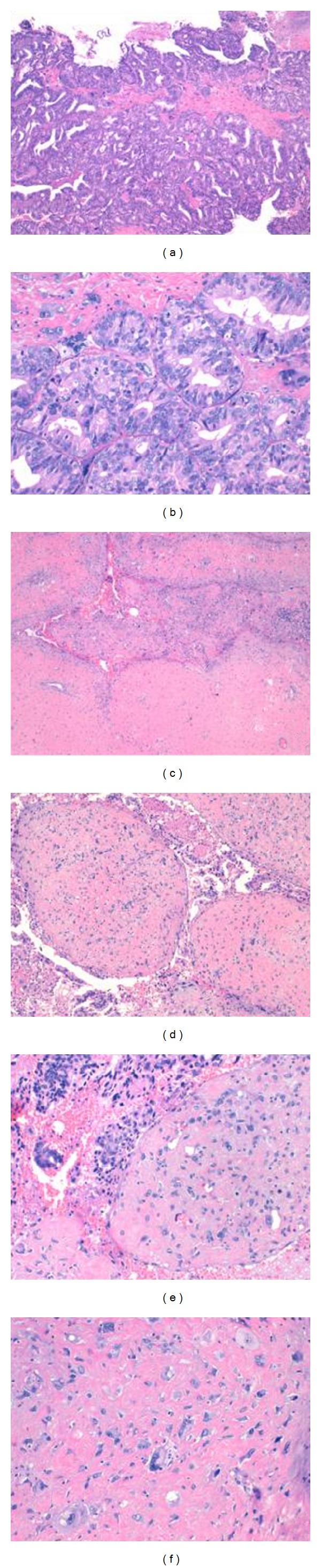
Microscopic finding. (a) Typical histopathologic aspect of prostatic ductal adenocarcinoma, showing a cribiform pattern. (b) Stroma with giant cells. (H-E, 50x–100x). (c, d, and e) Pleomorphic sarcoma characterized to have overall glandular growth pattern (H-E, 50x-100x-200x). (f) Malignant stroma showed increased cellularity and pleomorphism. Note: no spindled, fascicular, or heterologous growth pattern were presents. (H-E, 400x).

Standard H&E histologic sections demonstrated a biphasic tumour consisted of ductal adenocarcinoma (40%) and pleomorphic sarcoma (60%). Ductal adenocarcinoma was characterized by tall columnar cells with abundant amphophilic to eosinophil cytoplasm. Focal pseudostratified layers were also seen (Figure 2(a) and 2(b)). There were numerous atypical mitosis, cytological atypia, and areas of haemorrhage and necrosis. An admixed of cribriform and solid patterns were present. In subjacent stroma to epithelial areas, there were giant cells with large, irregular, and hyperchromatic nuclei (Figure 2(b)). The pleomorphic sarcoma was characterized to have overall glandular growth pattern, simulating a malignant phyllodes tumour (Figures 2(c)–2(f)). The malignant stroma showed increased cellularity, pleomorphism, and numerous atypical mitotic figures. No spindled, fascicular, or heterologous growth pattern were presents.

**Figure 3:**
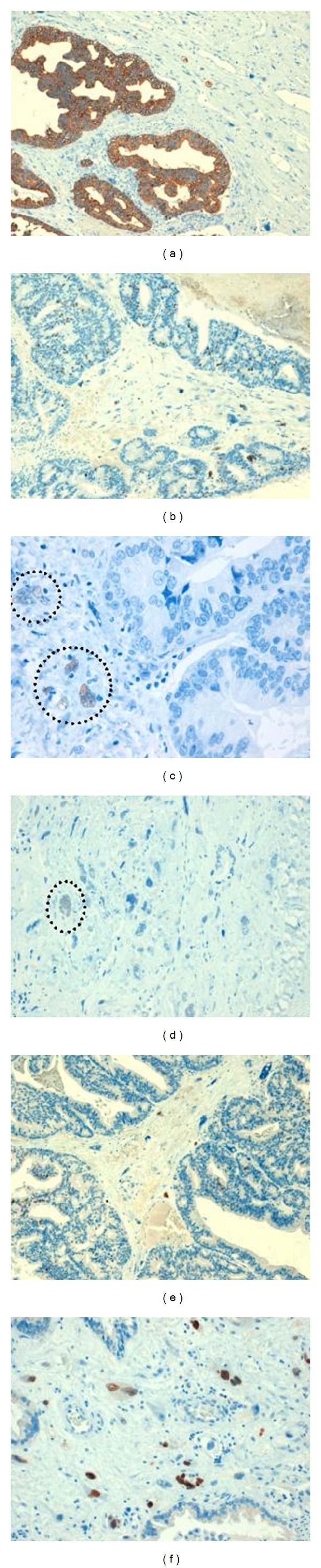
Immunohistochemical profile. (a) Cytokeratin AE1/ae3 stain showing a strong reactivity in epithelial component and focal expression is present at mesenchymal component (DAB, 100x). (b) Ki-67 nuclear positivity in both epithelial and mesenchymal component (DAB, 100x). (c and d) Estrogen and progesterone receptor expression. Multinucleated stromal cells showing nuclear positivity (Circle) (DAB, 400x). (e and f) p63 immunoreactivity in epithelial and stromal elements (DAB, 100x-200x).

Immunohistochemistry studies showed a focal expression to cytokeratin AE1/AE3 in stromal component (Figure 3(a)). Proliferate index expression was moderate in both components (Figure 3(b)). Estrogen and progesterone receptors showed nuclear immunostaining in mesenchymal multinucleated giant cells, with no reactivity at the epithelial component (Figures 3(c) and 3(d)). A nuclear reactivity was observed for p63 in both neoplastic epithelial and mesenchymal component (Figures 3(e) and 3(f)). Ductal adenocarcinoma showed expression for PSA and P504. Both neoplastic epithelial and mesenchymal components exhibited no staining for smooth muscle actin,  desmin,  S-100 protein, CD34, and *β*-GCH.

## 5. Discussion

Sarcomatoid carcinoma, also termed carcinosarcoma and spindle-cell carcinoma, is a rare biphasic malignancy in the prostate [1–3]. The two elements of SC are a malignant epithelial (carcinomatous) component and a malignant mesenchymal (sarcomatous) component with the presence or absence of heterologous elements [5–7]. The origin of these tumours has been controversial. It is the consideration of some investigators that SC is merely a collision of sarcoma and carcinoma which develop independently in the prostate. Other investigators however suggested that both components arise from an omnipotent cell [6, 11, 12]. In a study using loss-of-heterozygosity analysis in sarcomatoid prostatic adenocarcinoma, an evidence that the carcinomatous and sarcomatous elements are clonally related supported the hypothesis that a single malignant process underlies the aetiology of sarcomatoid carcinoma of the prostate [10]. Regardless its origin, the most recent World Health Organization classification of urinary tract tumours does not distinguish between SC and CS and use the term “sarcomatoid carcinoma” to denote all of these lesions [1].

Previous studies have showed a prior history of acinar prostatic adenocarcinoma in 48% and 65% of patients with sarcomatoid prostatic carcinoma [6, 7], and these findings support, in these cases, a progression of the prior acinar adenocarcinoma to a higher-grade tumour. In our case, there was no previous history of prostatic neoplasia, and no acinar adenocarcinoma was demonstrated. However, a ductal prostatic carcinoma was present at the initial diagnostic, and this variant is considered a higher-grade epithelial tumour. Additionally, a low percentage (10%) of SCs show ductal prostatic adenocarcinoma as part of epithelial component in prostatic SC [7].

Microscopically, the carcinomatous and sarcomatous components are admixed, with blending of the two in some areas. Previous studies showed two kinds of sarcomatous elements: undifferentiated spindled type and heterologous type [5–7, 12]. The majority of SC demonstrated an undifferentiated spindled appearance type (76%) and the rest of the sarcomatoid component are heterologous type (24%), that resembled osteosarcoma, chondrosarcoma, and rhabdomyosarcoma [5–7]. In our case, sarcomatoid component showed a homologous-like appearance, simulating a malignant phyllodes tumour, characterized by stromal overgrowth, cellularity, atypical stromal cells, infiltrative pattern, and necrosis. To our knowledge, this sarcomatoid component has not been previously informed. Independently of the sarcomatoid-type finding, the prognosis seems similar.

By immunohistochemistry, epithelial elements react with cytokeratins, PSA, and PSAP, whereas sarcomatoid elements react with vimentin or specific markers corresponding to the mesenchymal differentiation if present [5–7]. In our case, we could demonstrate expression for cytokeratin AE1/AE3, in both components but not PSA and P540 (only positive at epithelial cells). Additionally, we obtained expression for progesterone and estrogen receptors at mesenchymal pleomorphic cells. This finding has been described in stromal tumours of uncertain malignant potential (STUMP) and stromal sarcoma [13]. Probably in our tumour, the hormone expression at mesenchymal cells confirm a homologous differentiation as stromal sarcoma-like. Another immunohistochemical finding was the nuclear expression in epithelial and mesenchymal component for p63. In spindle cell lesions of the urinary bladder, expression of several cytokeratins with p63 could help to the diagnostic of sarcomatoid carcinoma [14]. In our case, the expression of cytokeratin and p63, coupled within the context of morphology, help to reach final diagnostic.

Due to the limited experience, there are no standard treatment recommendations the management of CSs of the prostate. Operable tumours are treated with surgery [6, 7]. Surgeries with curative intent include radical retropubic prostatectomy, radical cystoprostatectomy, suprapubic prostatectomy, and pelvic exenteration [6, 7]. Patient with SC have poor prognosis, with an actual risk of death of 20% within one year of diagnosis. In fact, nonsurgical therapy (androgen ablation treatment and chemotherapy) seems to be ineffective, and 55.5% of patients are unresponsive to chemotherapy (taxotere, estramustine, carboplatinum, or cisplatinum) [7]. In conclusion, SC of the prostate is an exceedingly rare tumour. Retrospective analyses render prostate SC as one of the most aggressive prostate malignancies. The prognosis is dismal regardless of other histologic or clinical findings.

## Figures and Tables

**Table 1:** Antibodies used for immunohistochemical studies.

Antigen	Clone	Dilution	Pretreatment	Tumour cell reactivity	Source
Ki-67	MIB1	Prediluted	pH6	Nuclear	Dako, Carpinteria, Calif, USA
ER	1D5	1/35	pH6	Nuclear stromal cells	Dako, Carpinteria, Calif, USA
PR	PGR36	1/70	pH6	Nuclear stromal cells	Dako, Carpinteria, Calif, USA
P-504	I3H4	Prediluted	pH9	Epithelial cells	Dako, Carpinteria, Calif, USA
PSA	ERPR8	Prediluted	pH6	Epithelial cells	Dako, Carpinteria, Calif, USA
CK AE1/AE3	AE1/AE3	Prediluted	pH6	Both components	Dako, Carpinteria, Calif, USA
SMA	HHF35	Prediluted	pH6	Negative	Dako, Carpinteria, Calif, USA
S-100	Polyclonal	1/3000	pH6	Negative	Dako, Carpinteria, Calif, USA
CD34	QBEND-10	Prediluted	pH6	Negative	Dako, Carpinteria, Calif, USA
p63	4A4	Prediluted	pH6	Nuclear	Dako, Carpinteria, Calif, USA
*β*-GCH	Polyclonal	Prediluted	pH9	Negative	Dako, Carpinteria, Calif, USA
